# Unveiling the hidden clues: Döhle body-like inclusions as morphological markers for MYH9-related disorders: A case report

**DOI:** 10.1097/MD.0000000000036735

**Published:** 2023-12-22

**Authors:** Yan Zhang, Zhongbao Zuo, Wenyan Yu, Aifang Xu

**Affiliations:** a Department of Clinical Laboratory, Xixi Hospital of Hangzhou, Hangzhou, Zhejiang, China.

**Keywords:** Döhle body-like inclusions, giant platelet, MYH9-RDs, thrombocytopenia

## Abstract

**Rationale:**

This study aimed to address the diagnostic challenges associated with MYH9-related disorders (MYH9-RDs) and highlight the importance of recognizing Döhle body-like inclusions as crucial diagnostic markers for this condition.

**Patient concerns:**

Patients with MYH9-RDs often present with mild and diverse clinical characteristics, leading to misdiagnosis, delayed diagnosis, and inappropriate treatments, such as hormonal therapy and splenectomy. This section highlights the significance of understanding atypical clinical presentations and their impact on patients’ well-being.

**Diagnoses:**

This section emphasizes the misdiagnosis of MYH9-RDs as immune thrombocytopenia due to overlapping clinical features. This highlights the need for a comprehensive approach, including detailed personal and family history, careful review of peripheral blood smears, and identification of Döhle body-like inclusions to differentiate MYH9-RDs from other conditions.

**Intervention:**

This study advocates for a shift in the diagnostic approach, urging physicians to pay closer attention to the morphological features observed in peripheral blood smears, particularly the presence of Döhle body-like inclusions and large platelets. This emphasizes the importance of avoiding unnecessary diagnostic studies through effective utilization of this simple and reliable method.

**Outcomes:**

By adopting a comprehensive approach that combines gene sequencing with morphological analysis, an accurate diagnosis of MYH9-RDs can be achieved. Early identification of MYH9-RDs allows for appropriate management strategies, genetic counseling, and prevention of complications associated with the condition.

**Lessons:**

This section highlights the lessons learned from this study, emphasizing the need for increased awareness among healthcare professionals about MYH9-RDs and the importance of incorporating peripheral blood smear evaluations into the diagnostic process. This emphasizes the significance of accurate diagnosis to prevent unnecessary treatments and ensure appropriate patient care.

## 1. Introduction

MYH9-related disorders (MYH9-RDs) encompass a spectrum of rare genetic conditions characterized by diverse clinical presentations and isolated thrombocytopenia. These disorders are caused by mutations in MYH9, which encodes the non-muscle myosin heavy chain IIA protein.^[1]^ The clinical manifestations of MYH9-RDs vary widely, ranging from mild cases of isolated thrombocytopenia to more severe forms associated with progressive nephropathy, hearing loss, and cataracts.^[2]^

The diagnostic journey for patients with MYH9-RDs can be challenging due to overlapping features with other thrombocytopenic disorders, particularly immune thrombocytopenia (ITP).^[3]^ Misdiagnosis of MYH9-RDs as ITP is common, leading to inappropriate treatments and delayed identification of the underlying condition. Moreover, the lack of awareness and limited understanding of MYH9-RDs among healthcare professionals further contributes to the diagnostic complexities.

In recent years, the identification of morphological clues in peripheral blood smears has emerged as a valuable diagnostic strategy for identifying MYH9-RDs. Notably, the presence of Döhle body-like inclusions and large platelets has been observed in patients with MYH9-RDs, providing important morphological markers for differentiating these disorders from other thrombocytopenic conditions.

This article aims to shed light on the diagnostic challenges associated with MYH9-RDs and to emphasize the significance of recognizing Döhle body-like inclusions as crucial diagnostic markers for this condition. We explored the clinical complexities, consequences of misdiagnosis, and implications for patient care. Additionally, we discuss the importance of adopting a comprehensive approach that combines gene sequencing with morphological analysis, highlighting the role of peripheral blood smear evaluation in achieving accurate diagnoses and facilitating appropriate management strategies.

By enhancing the understanding of MYH9-RDs and promoting awareness among healthcare professionals, we aimed to improve diagnostic accuracy, minimize misdiagnosis, and enhance patient outcomes.

## 2. Case presentation

**Figure 1.**
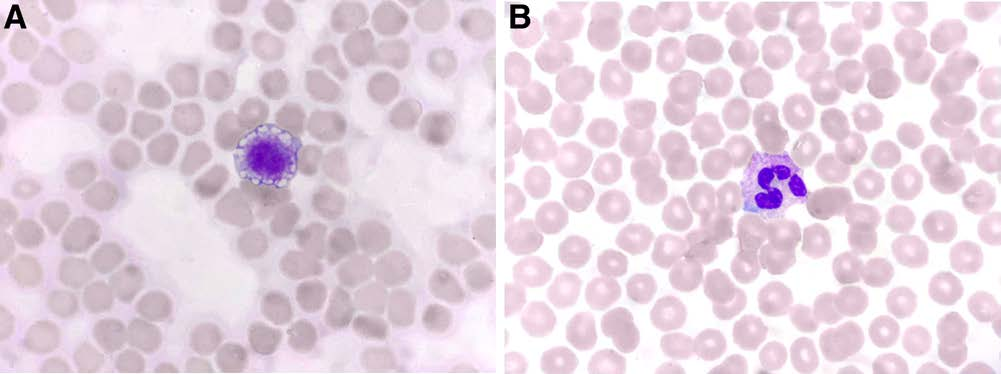
Peripheral smear containing a gaint platelet (A) and Döhle-like body (B).

Based on the information provided, a 26-year-old man presented with frequent ecchymosis (bruising) and was found to have thrombocytopenia (platelet count = 22 × 10^9^/L). Further laboratory investigations confirmed macrothrombocytopenia (presence of abnormally large platelets) and Döhle body-like inclusions in the blood smear (Fig. 1). These findings strongly suggest a diagnosis of MYH9-RDs.

The patient’s clinic1al history reveals a long-standing history of frequent nasal bleeding and bruising since childhood. Previous visits to multiple hospitals failed to identify the exact cause of the thrombocytopenia. Additionally, the patient has been experiencing hematuria (blood in urine) since the age of 20 years.

Laboratory tests revealed unremarkable results for coagulation function, clotting factors, blood urea nitrogen, creatinine, alanine aminotransferase, and aspartate aminotransferase levels. The maximum amplitude value, indicative of platelet aggregation, was within the normal range. A platelet antibody IgG test yielded a negative outcome. Serum measurements indicated total bilirubin and unconjugated bilirubin concentrations of 26.33 µmol/L (reference range: 0–23 µmol/L) and 15.7 µmol/L (reference range: 0–13.7 µmol/L), respectively.

**Figure 2.**
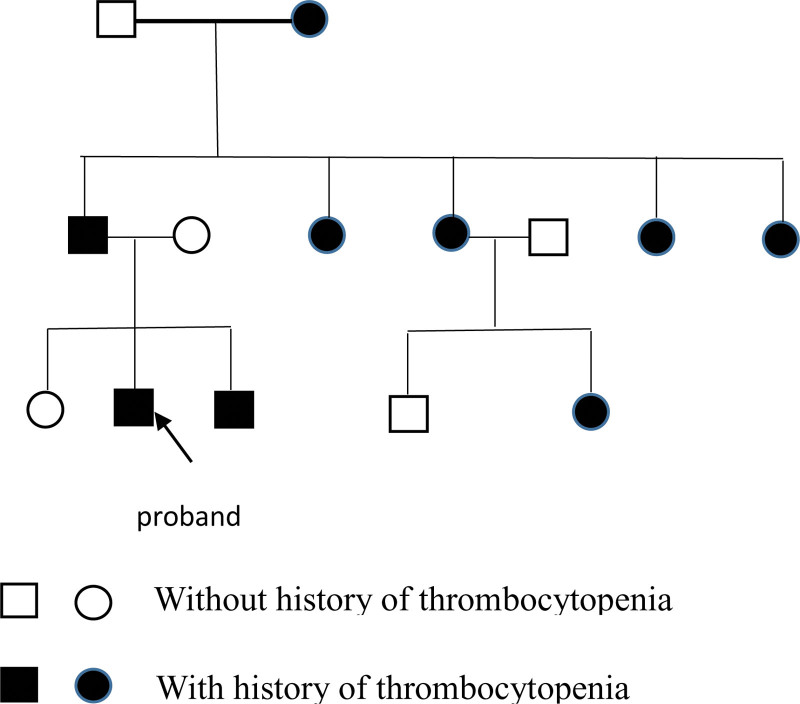
Family pedigree. The arrow indicates the proband. □, ○: without history of thrombocytopenia. , ●: with history of thrombocytopenia.

The detailed family history sheds more light on the hereditary nature of thrombocytopenia in this family and further supports the diagnosis of MYH9-RDs. The following are some key points regarding family history and the implications for the diagnosis:

*Multigenerational inheritance* (Fig. 2): The presence of thrombocytopenia or bleeding episodes in the patient’s grandmother, father, 4 aunts, younger brother, and 1 cousin strongly suggested a hereditary pattern of thrombocytopenia within the family. This pattern of inheritance is consistent with that of MYH9-RDs, which are known to be inherited in an autosomal dominant manner.

*Clinical features in family members*: The father had thrombocytopenia and exhibited a higher mean platelet volume and platelet distribution width than normal, indicating larger platelets. This is in line with the macrothrombocytopenia observed in MYH9-RDs. The father also presented with isolated proteinuria, which can be associated with MYH9-RDs, suggesting potential kidney involvement.

*Clinical features in the younger brother*: The younger brother experienced severe nosebleeds and persistent bleeding, requiring platelet transfusions. The fluctuating platelet count (brief increase followed by a decrease) was consistent with the characteristic variability observed in MYH9-RDs. This highlights the variable expressivity of disorders within the same family.

*Cousin’s bleeding episode*: One of the cousins experienced bleeding related to childbirth, although the specific treatment measures are unknown. This highlights the potential bleeding tendency of individuals with MYH9-RDs, which can pose challenges during pregnancy and childbirth.

**Figure 3.**
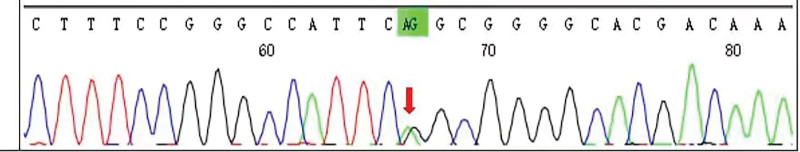
Patient’s gene sequencing results (the red arrow indicates genetic variation sites).

*Genetic testing results* (Fig. 3): The patient’s genetic testing revealed a mutation in exon 41 of the MYH9 gene (c.5797C>T, p.R1933*), providing a definitive confirmation of the diagnosis of MYH9-RDs in the patient. This result further supports the hereditary nature of the disorder in the family.

*Deafness and MYH9-RDs*: The patient’s grandmother had mild deafness, and there is a suspicion that it may be related to MYH9-RDs. However, distinguishing whether deafness in older individuals with MYH9-RDs is directly caused by gene mutation or is a separate age-related condition can be challenging. Although deafness can occur in MYH9-RDs, further evaluation and genetic studies are needed to establish a clear association.

## 3. Discussion

MYH9-RDs encompass an autosomal dominant inherited condition characterized by thrombocytopenia, giant platelets, and Döhle body-like inclusions.^[4]^ These Döhle body-like inclusions can be observed in the cytoplasm of neutrophils, monocytes, eosinophils, and basophils in peripheral blood smears.^[5]^ However, clinical misdiagnosis and oversight are common because of the tendency to overlook these inclusions, especially when they are faintly stained, and when laboratory personnel lack attentiveness and thoroughness, leading to missed diagnoses. Additionally, MYH9-RDs are rare disorders in real-life scenarios, which has resulted in some laboratory staff confusing the Döhle body-like inclusions with true Döhle bodies.

Morphologically, true Döhle bodies are small, often round or oval structures with indistinct cloud-like borders. In contrast, the Döhle body-like inclusions in MYH9-RDs were distributed along the cellular periphery, exhibiting clear features and well-defined, tangible boundaries. It is important to note that while true Döhle bodies manifest during infections and are typically accompanied by granulocyte toxic granules, vacuolar degeneration, nuclear condensation, and other signs of granulocyte poisoning, they generally do not coincide with the presence of giant platelets. Furthermore, true Döhle bodies tend to disappear as the infection is brought under control, whereas Döhle body-like inclusions persist throughout an affected individual’s lifetime. Therefore, a meticulous examination of blood smears is valuable and straightforward for evaluating suspected cases of thrombocytopenia.

Genetic analysis of MYH9 confirmed the presence of a heterozygous mutation (c.5797C>T, p.R1933*) in exon 41 of the patient. These clinical and laboratory findings were consistent with a diagnosis of MYH9-RDs. When clinical assessments and laboratory tests indicate hereditary thrombocytopenia in patients and their families, genetic testing becomes an indispensable tool for confirming diagnosis.

Bleeding symptoms in patients with MYH9-RDs exhibit heterogeneity. While some patients do not experience significant bleeding tendencies, others may present with mild to moderate symptoms such as easy bruising, recurrent epistaxis, gingival bleeding, and increased menstrual bleeding. The bleeding tendency of MYH9-RDs is primarily associated with the degree of thrombocytopenia rather than platelet dysfunction. Notably, our patient’s platelet aggregation was within the normal range, consistent with the findings reported by Jiang et al.^[6]^

In addition to hematological changes, MYH9-RDs commonly present with various non-hematological disorders, and the severity and manifestation of these conditions can vary over time. Approximately 50% of patients experience hearing impairment, nephropathy or proteinuria in 25% of cases, and bilateral cataracts are observed in approximately 20% of cases^.[1,7,8]^

It is important to recognize that different individuals within the same family may exhibit varying phenotypes at different ages, as observed in our patient’s family. Furthermore, the phenotype of a patient with an MYH9 mutation can evolve over time, as supported by previous research.^[9]^ Progressive manifestations that can develop over time include neurological deafness, senile cataracts, and nephritis, which may ultimately progress to end-stage renal disease.^[10,11]^ In the case of our patient and his father, persistent hematuria and proteinuria were observed, necessitating regular monitoring of the kidney function.

The patient in this family study presented with subtle and diverse clinical characteristics that were easily overlooked, particularly when historical platelet counts and a complete family history were not readily available. The patient’s isolated thrombocytopenia necessitated a prolonged search for the underlying cause. ITP, a common condition characterized by isolated thrombocytopenia, can often lead to the misdiagnosis of relatively rare MYH9-RDs. The negative result for platelet antibody IgG suggests the absence of specific antibodies against platelets, which is important in ruling out certain conditions, such as immune thrombocytopenia. In fact, many patients with MYH9-RDs have been misdiagnosed with ITP, resulting in inappropriate treatments such as hormonal therapy and splenectomy.^[4,12]^ A study based on the Italian registry reported that 60% of cases were initially diagnosed as ITP, with 30% of patients receiving inappropriate treatments.^[13]^ Therefore, physicians should pay close attention to detailed personal and family histories and conduct a thorough review of peripheral blood smears, especially when Döhle body-like inclusions are observed alongside large platelets, as this strongly indicates the presence of MYH9-RDs. Due to the rarity of this condition and lack of awareness, MYH9-RDs are often misdiagnosed, leading to unnecessary diagnostic procedures. Evaluation of a peripheral blood smear is a simple and effective method for accurate diagnosis. This case highlights the importance of a comprehensive approach that combines gene sequencing with morphological analysis (thrombocytopenia, giant platelets, and Döhle-like bodies), particularly in identifying Döhle body-like inclusions and true Döhle bodies, which helps differentiate the underlying causes of long-standing thrombocytopenia.

## 4. Conclusion

The diagnosis of inherited thrombocytopenias with nonspecific clinical presentations poses a significant challenge. A comprehensive approach involving clinical evaluation, careful examination of blood smears, and genetic testing is crucial for the diagnosis of MYH9-RDs. We emphasize the importance of recognizing the morphological characteristics of the Döhle body-like inclusions as a valuable diagnostic clue. Acquiring the ability to identify laboratory indicators of inherited thrombocytopenia is crucial for preventing misdiagnosis.

## Author contributions

**Conceptualization:** Yan Zhang, Aifang Xu.

**Data curation:** Yan Zhang, Zhongbao Zuo, Wenyan Yu.

**Investigation:** Wenyan Yu.

**Writing – original draft:** Yan Zhang.

**Writing – review & editing:** Zhongbao Zuo, Aifang Xu.
